# Year-round spatiotemporal distribution of harbour porpoises within and around the Maryland wind energy area

**DOI:** 10.1371/journal.pone.0176653

**Published:** 2017-05-03

**Authors:** Jessica E. Wingfield, Michael O’Brien, Vyacheslav Lyubchich, Jason J. Roberts, Patrick N. Halpin, Aaron N. Rice, Helen Bailey

**Affiliations:** 1 Chesapeake Biological Laboratory, University of Maryland Center for Environmental Science, Solomons, Maryland, United States of America; 2 Marine Geospatial Ecology Laboratory, Nicholas School of the Environment, Duke University, Durham, North Carolina, United States of America; 3 Biacoustics Research Program, Cornell Lab of Ornithology, Cornell University, Ithaca, New York, United States of America; Institute of Deep-sea Science and Engineering, Chinese Academy of Sciences, CHINA

## Abstract

Offshore windfarms provide renewable energy, but activities during the construction phase can affect marine mammals. To understand how the construction of an offshore windfarm in the Maryland Wind Energy Area (WEA) off Maryland, USA, might impact harbour porpoises *(Phocoena phocoena*), it is essential to determine their poorly understood year-round distribution. Although habitat-based models can help predict the occurrence of species in areas with limited or no sampling, they require validation to determine the accuracy of the predictions. Incorporating more than 18 months of harbour porpoise detection data from passive acoustic monitoring, generalized auto-regressive moving average and generalized additive models were used to investigate harbour porpoise occurrence within and around the Maryland WEA in relation to temporal and environmental variables. Acoustic detection metrics were compared to habitat-based density estimates derived from aerial and boat-based sightings to validate the model predictions. Harbour porpoises occurred significantly more frequently during January to May, and foraged significantly more often in the evenings to early mornings at sites within and outside the Maryland WEA. Harbour porpoise occurrence peaked at sea surface temperatures of 5°C and chlorophyll *a* concentrations of 4.5 to 7.4 mg m^-3^. The acoustic detections were significantly correlated with the predicted densities, except at the most inshore site. This study provides insight into previously unknown fine-scale spatial and temporal patterns in distribution of harbour porpoises offshore of Maryland. The results can be used to help inform future monitoring and mitigate the impacts of windfarm construction and other human activities.

## Introduction

With the development of offshore energy infrastructure and increases in ship traffic, the world’s oceans are becoming busier and noisier [1, 2]. Noisier oceans are a concern for marine mammals as they use sound for communication, foraging, and navigation [3, 4]. Increased background noise from ships, dredging, pile-driving, seismic surveys and other anthropogenic sources has caused a variety of behavioural responses in many cetacean species [5–9]. It is critical to understand the fine-scale spatial and temporal distribution of cetaceans in areas of planned developments, like offshore windfarms, in order to inform regulators and developers on how to most effectively avoid and minimize negative impacts during the construction phase when loud sounds may be emitted.

Over the last decade there has been rapid development of offshore wind energy off the coast of the United Kingdom and elsewhere in Europe [10]. Disturbance to cetaceans may occur during pile-driving of the wind turbine foundation and in response to increased vessel traffic associated with the construction [11]. The harbour porpoise, *Phocoena phocoena*, is the most common and widely distributed cetacean in European waters [12], and has therefore been the focus of many studies on the effects of offshore wind turbine construction. The responses of porpoises to windfarms have varied depending on the life-cycle phase of the windfarm (construction, early operation, long-term operation). Harbour porpoises decreased their acoustic activity for up to 24 hours at a distance of 18 km from the Danish Horns Rev II windfarm following pile-driving [13]. A study conducted during the operational phase of an offshore windfarm in the Dutch North Sea found that porpoise presence had increased in the area, possibly due to an increase in fish or the absence of fishing vessels [14]. In contrast, Teilmann & Carstensen [15] observed a significant decline in porpoise echolocation activity from 2003 to 2012 relative to baseline levels in 2001 and 2002 inside a windfarm constructed in 2002–2003 in the Danish western Baltic Sea. However, there was a significant increase in encounter rate and echolocation activity in 2011 and 2012 relative to previous years (2003–2009) [15].

**Fig 1:**
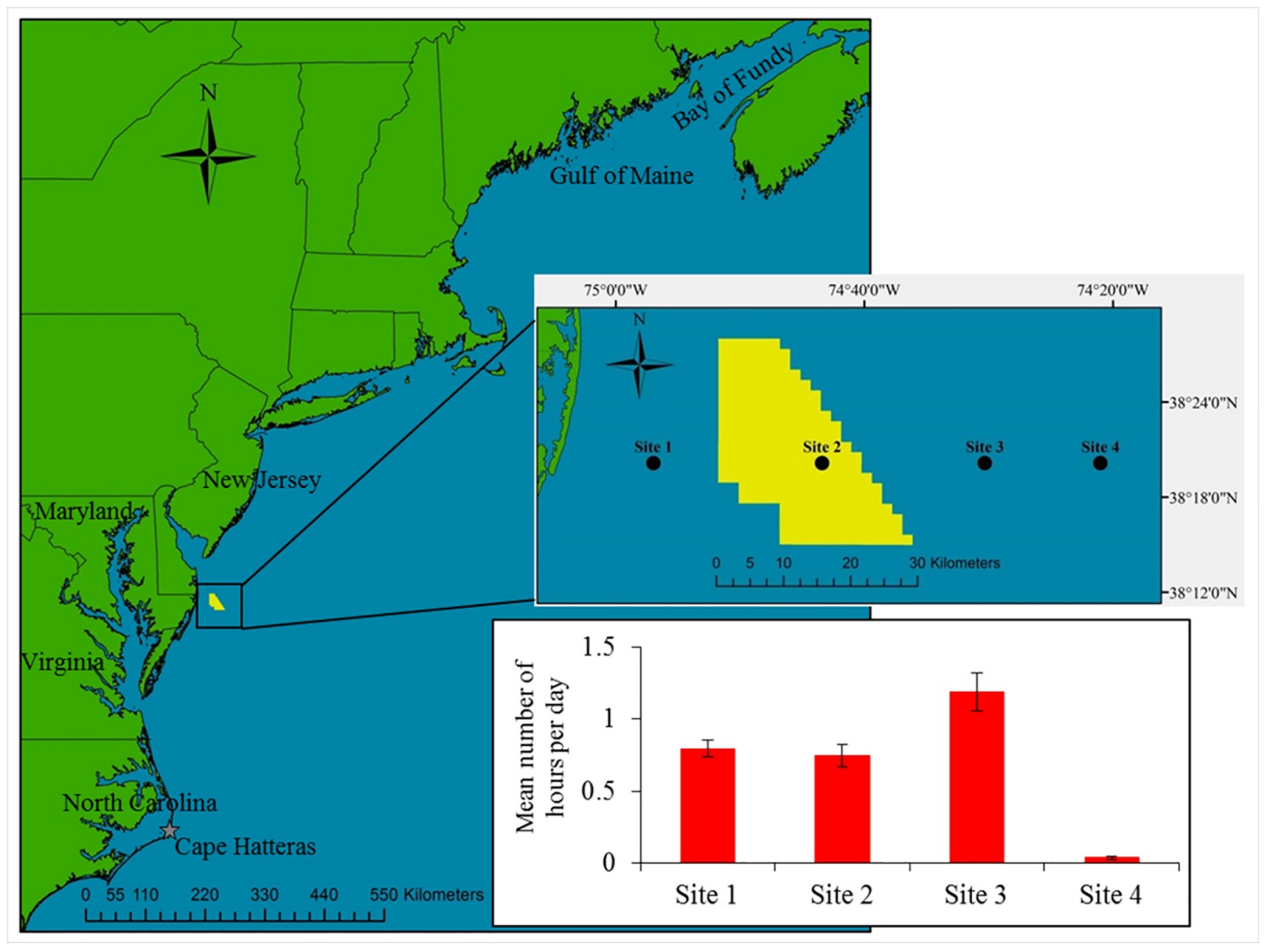
Map of the northeastern coast of the United States and study location. Displayed is the Maryland Wind Energy Area (yellow) and the four C-POD sites (inset). Bar plot shows the mean (± SE) number of hours per day that porpoises were detected throughout the study period.

The Maryland Wind Energy Area (WEA) is located 20 to 40 km offshore of Maryland in the northwestern Atlantic, and is approximately 324 km^2^. The Gulf of Maine/Bay of Fundy population of harbour porpoises in the northwestern Atlantic consists of approximately 80,000 individuals [16, 17]. They do not appear to follow a specific migratory route nor do they have a temporally coordinated migration, but they typically occur off New Jersey to North Carolina in winter (January to March), and from the Bay of Fundy to New Jersey in spring, summer, and fall [18–21] (Fig 1). Annually, an estimated 709 harbour porpoises from this stock are incidentally bycaught in fisheries in US and Canadian waters [17]. Fisheries bycatch is considered one of the single greatest threats facing marine mammals in the United States [22]. Despite there being a number of both aerial and boat-based visual surveys conducted offshore of Maryland, harbour porpoises have been sighted very few times [23, 24]. Year-round distribution of harbour porpoises off Maryland is therefore not well understood. In an attempt to increase understanding of cetacean distribution off the east coast of the United States, Roberts et al. [25] developed a habitat-based density model using aerial and boat-based sightings data to predict year-round harbour porpoise densities. Despite there being no sightings south of New Jersey included in the model, the predictions extend to Cape Hatteras, North Carolina. However, the few porpoise sightings reported off Maryland and strandings on beaches in North Carolina, justified the model’s extension southwards beyond New Jersey [24, 26, 27].

Here, we aimed to characterize year-round patterns in harbour porpoise occurrence and foraging behaviour in relation to temporal and environmental variables within and around the Maryland WEA. Data were collected using passive acoustic devices called C-PODs, which detect and log cetacean echolocation clicks (Chelonia Ltd., UK). C-PODs, and their predecessor, T-PODs, have been widely used to detect both dolphins and porpoises [11, 13, 28–31]. Harbour porpoises can be difficult to observe during boat-based or aerial surveys because of their small size and elusive behaviour [32]. Visual detectability of harbour porpoises significantly declines in sea states of Beaufort 2 or higher, limiting the number of reliable sightings available for abundance estimates [33, 34]. Therefore, passive acoustic monitoring is a cost-effective alternative for detecting porpoises in any sea state and at any time of day. Passive acoustic devices also allow for the collection of continuous and long-term occurrence data, and therefore provide a useful tool for validating patterns of relative abundance from habitat-based predictive models [35, 36]. The acoustic data collected in this study were also used to evaluate the accuracy of Roberts et al.’s [25] habitat-based density predictions for harbour porpoises offshore of Maryland.

## Materials and methods

### Data collection and processing

The Maryland WEA is located approximately 20–40 km offshore of Ocean City, Maryland, USA (Fig 1). The substrate within and around the WEA is predominantly sand [37]. The eastern edge of the WEA has high ship traffic, where ships pass as they approach or exit the Delaware Bay [38]. Passive acoustic monitoring of marine mammals in the area began in November 2014 to obtain baseline data prior to windfarm construction. C-PODs (Version 1, Chelonia Ltd., UK) were deployed at four sites up to 63 km offshore, including within and up to 35 km outside of the WEA to detect harbour porpoises (*Phocoena phocoena*) (Fig 1). Moorings were bottom-anchored, with the C-POD positioned approximately 5 m from the sea floor, in approximately 20–45 m water depth. C-PODs were recovered and re-deployed approximately every three months. Data in this study extend to May 2016. C-PODs continuously monitor the 20–160 kHz frequency range, logging the center frequency, frequency trend, duration, intensity, and bandwidth of tonal clicks. High-frequency harbour porpoise clicks, which have a peak frequency of approximately 131 kHz and range from 110 to 180 kHz [39, 40], can be detected by a C-POD from several hundred meters away [41]. The KERNO classifier within the CPOD.exe software (Chelonia Ltd., v. 2.044) then identifies click trains (sequences of at least 5 clicks) and assesses the likelihood of each click-train belonging to a dolphin or porpoise as either high (CetHi), medium (CetMed), or low (CetLow) [42]. A conservative approach was taken and only CetHi and CetMed harbour porpoise click trains were included in the analyses [31, 35]. A study combining T-POD detections (the predecessor of the C-POD) with visual observations determined the false detection rate to be very low indicating that the click train algorithm is efficient and conservative [43]. The data were exported and formatted to an hourly resolution with the number of minutes in each hour that a harbour porpoise click train was detected. Only hours with a complete 60 minutes of recording were used in the analyses.

### Temporal occurrence of porpoises

Temporal patterns in harbour porpoise detections were investigated for each site using generalized autoregressive moving average (GARMA) models [44]. This type of model accommodates non-Gaussian time-series data (e.g. auto-correlated count series), with potentially time-dependent covariates. The response variable in the model was the number of minutes per hour that harbour porpoises were detected and the explanatory variables were hour of the day (Eastern Standard Time, EST) and Julian day. To model cyclical annual and daily patterns, we applied two pairs of sinusoidal functions: sin(2*πt/d*) and cos(2*πt/d*), where period *d* is one day or one year, and *t* is the hour of the day or Julian day, respectively. Models were fit in the statistical software R [45] using the package gamlss.util [46]. Model selection was performed using the Akaike Information Criterion (AIC). Autocorrelation and partial autocorrelation plots were used to examine the remaining serial dependence (if any) in the final models’ residuals. Model fit was assessed by examining the residual plots [46].

### Foraging behaviour

A subset of the data was created that consisted of only hours during which harbour porpoises were detected. The C-POD custom software was used to calculate and export inter-click intervals (ICI), as the number of micro-seconds between clicks, for each click train detected. The inter-click intervals of harbour porpoise click trains have been found to vary in duration depending on the behaviour of the porpoise [47–49]. Click trains associated with foraging have lower ICIs and faster repetition rates than those associated with travelling [49, 50]. An ICI of 10 ms or less was used as the threshold to infer foraging [50–55]. Therefore, hours during which at least one of the ICIs was 10 ms or less were considered “foraging positive”, and hours with ICIs greater than 10 ms were deemed “foraging negative”. The presence/absence of foraging behaviour in each hour from the subset data was modeled for each site using generalized additive models (GAMs) with a binomial error distribution and logit link function [56]. The explanatory variables were hour of the day (EST) and Julian day. Due to the cyclical nature of the explanatory variables, a circular spline was used. Models were fit using the R package mgcv [57]. AIC was used to select the best model, and goodness of fit was evaluated using confusion matrices [58] and area under the receiver operating characteristic (ROC) curve [59]. Confusion matrices compare the binary predictions from the model to the observed presence/absence values [58], in this case the presence or absence of foraging. The closer the area under the ROC curve is to 1, the better the model fit [59]. Confusion matrices were calculated using the R package PresenceAbsence [60] and the area under the ROC curves was calculated using the package ROCR [61].

### Environmental data analysis

Using the full data set, the proportion of hours per week during which harbour porpoises were detected was compared to weekly median sea surface temperature (°C, SST), the natural log of chlorophyll *a* concentration (mg m^-3^), and fraction of the moon illuminated. Due to their small size and that they consume small forage-fish, it is difficult for harbour porpoises to retain large energy stores, and therefore they forage almost continuously with generally high capture success rates [62, 63]. Fine-scale distribution of forage-fish species is difficult to assess, but due to the attraction of these fish to areas of high primary productivity, chlorophyll *a* concentration can be used as a proxy for prey abundance [64, 65]. Although the degree of lunar illumination has been shown to affect the foraging behaviour of dolphins, its effect on porpoise foraging is not well studied [66]. Week numbers were assigned using the ISO week date standard (ISO-8601). Eight-day composites of SST (GOES Imager) and chlorophyll *a* concentration (Moderate Resolution Imaging Spectroradiometer (MODIS) onboard the Aqua satellite) were extracted for each day during the study period at each site using the NOAA Coastwatch tool Xtractomatic tool (http://coastwatch.pfel.noaa.gov/xtracto/) in R. Data on the fraction of the moon illuminated for each night were available from the Astronomical Applications Department of the US Naval Observatory (http://aa.usno.navy.mil/index.php). Weekly medians for each environmental variable were then calculated and compared to the proportion of hours per week with a harbour porpoise detection using a GAM with a Gaussian error distribution for each site (R package mgcv [57]). AIC was used for model selection, and the function gam.check within the mgcv package was used to assess goodness of fit by visualizing the model residuals [57]. Residual autocorrelation and partial autocorrelation plots were used to assess if any serial dependence remained uncaptured by the models.

### Comparison of acoustic data with habitat-based density predictions

Roberts et al. [25] developed habitat-based density models for several species of cetaceans, including harbour porpoises, off the US east coast using aerial and boat-based sightings data. A porpoise positive hour (PPH) is an hour during which the C-POD software identified at least one porpoise click train. Roberts et al.’s monthly density estimates [25] were compared with the median number of PPHs per day, total PPHs per month, maximum PPHs per day, and proportion of days per month harbour porpoises were present in the study area offshore of Maryland based on our acoustic detection data using Spearman’s rank correlation tests for each site [35, 36].

## Results

**Fig 2:**
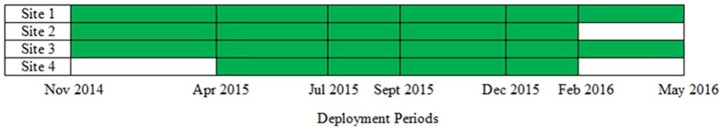
The C-POD deployment periods. Green indicates a complete, uninterrupted dataset and a blank space indicates there were no data during the corresponding deployment period, either due to instrument loss or malfunction.

C-PODs were deployed and recording for a median 521 days from 4^th^ November 2014 to 18^th^ May 2016 (Table 1). Instrument loss and malfunction resulted in some data gaps at sites 2 and 4 (Fig 2). Harbour porpoises were detected during the greatest proportion of days at the most inshore site, site 1, but were detected for the most hours at the farther offshore site, site 3 (Table 1).

**Table 1:** Summary of the harbour porpoise acoustic data collected at each of the four sites offshore of Maryland.

Site	Recording period	Distance offshore (km)	# of recording days	% of days present	% of hours present	Maximum # of minutes per hour with a detection
1	4^th^ November 2014 to 18^th^ May 2016	12	562	36.8	3.2	17
2	5^th^ November 2014 to 28^th^ February 2016	30	481	27.0	3.1	14
3	4^th^ November 2014 to 17^th^ May 2016	50	561	26.9	5.0	43
4	23^rd^ April 2015 to 27^th^ February 2016	63	311	3.5	0.2	4

### Temporal occurrence of harbour porpoises

**Fig 3:**
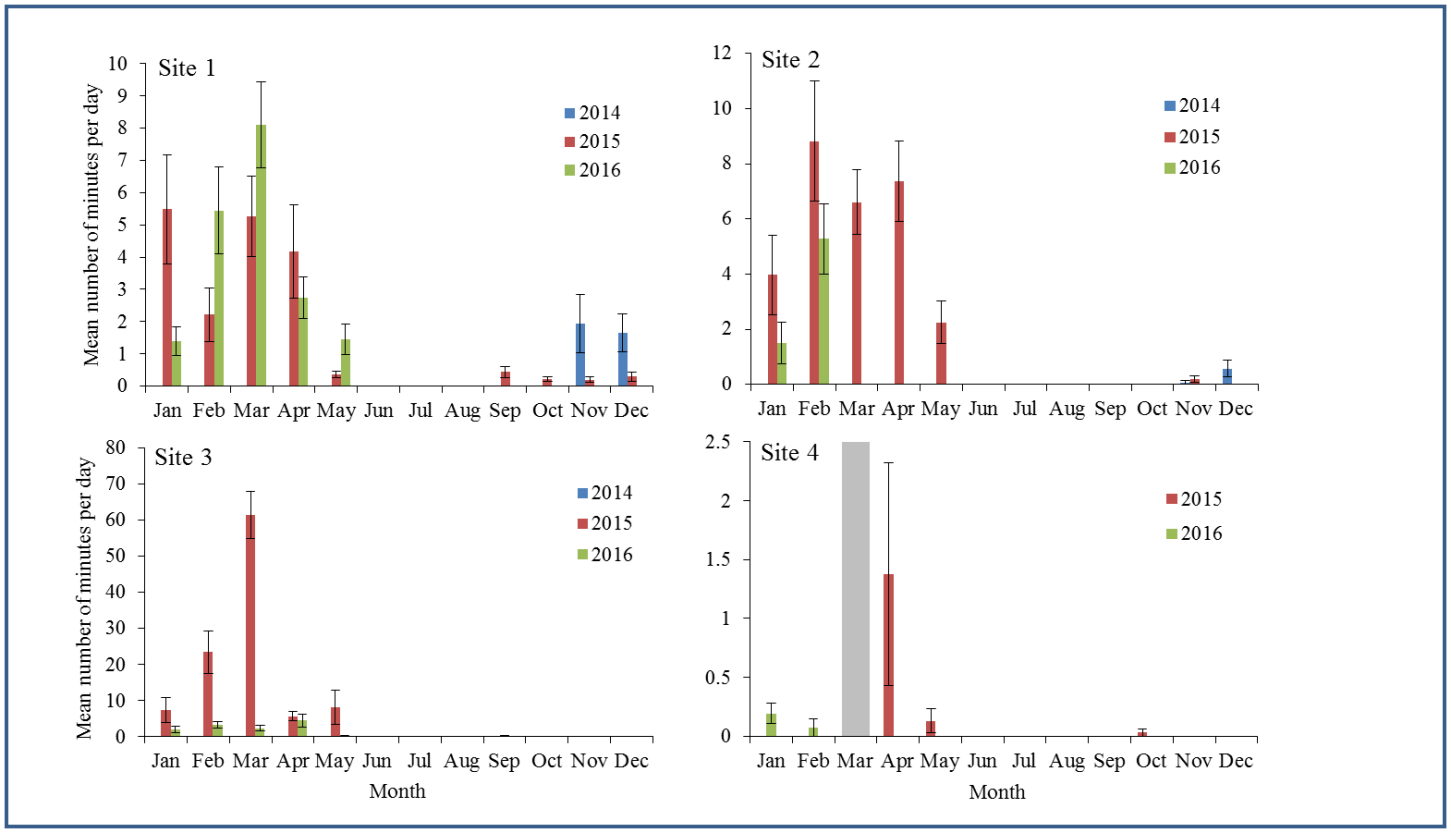
The mean (± SE) number of minutes per day during which harbour porpoises were acoustically detected at each site offshore of Maryland. There were no data in March at site 4 due to instrument loss.

**Fig 4:**
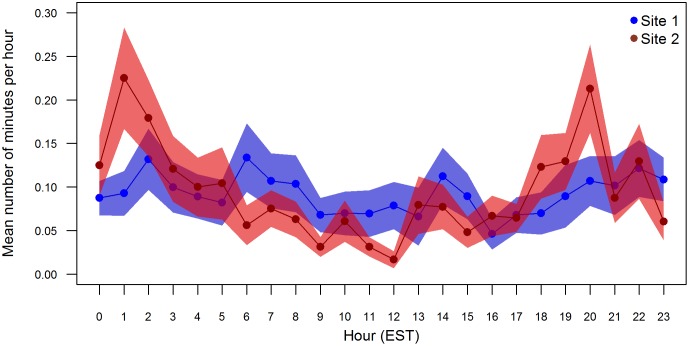
The mean number of minutes in each hour that harbour porpoises were detected at sites 1 and 2, where the hour of the day was a significant factor in the generalized auto-regressive moving average (GARMA) models of hourly porpoise presence. The shaded polygons represent the standard error.

A Poisson inverse-Gaussian distribution yielded the lowest AIC scores for GARMA models of the temporal patterns in harbour porpoise presence at all sites except site 4. The Poisson inverse-Gaussian distribution is well suited to handle extra-Poisson variation and has been used in a variety of disciplines [67]. A zero-inflated Poisson distribution yielded the lowest AIC score for the GARMA model of the data at site 4. Julian day was retained in all final models as a significant predictor for the number of minutes harbour porpoises were detected in an hour (S1 Table). Harbour porpoises were present significantly more often during the winter and spring months (January to May), and rarely in the summer and fall (Fig 3). There was a high degree of inter-annual variability in the number of minutes harbour porpoises were detected per day (Fig 3). There were more detections at site 1 in 2016 than in 2015, but more detections at sites 2 and 3 in 2015 compared to 2016 (Fig 3). The hour of the day was retained as a significant factor in the GARMA models for sites 1 and 2 only (S1 Table). A particularly strong diel pattern was seen at site 2 with peaks in occurrence at 01:00 and 20:00 and lowest occurrence at noon (Fig 4).

### Foraging behaviour

**Fig 5:**
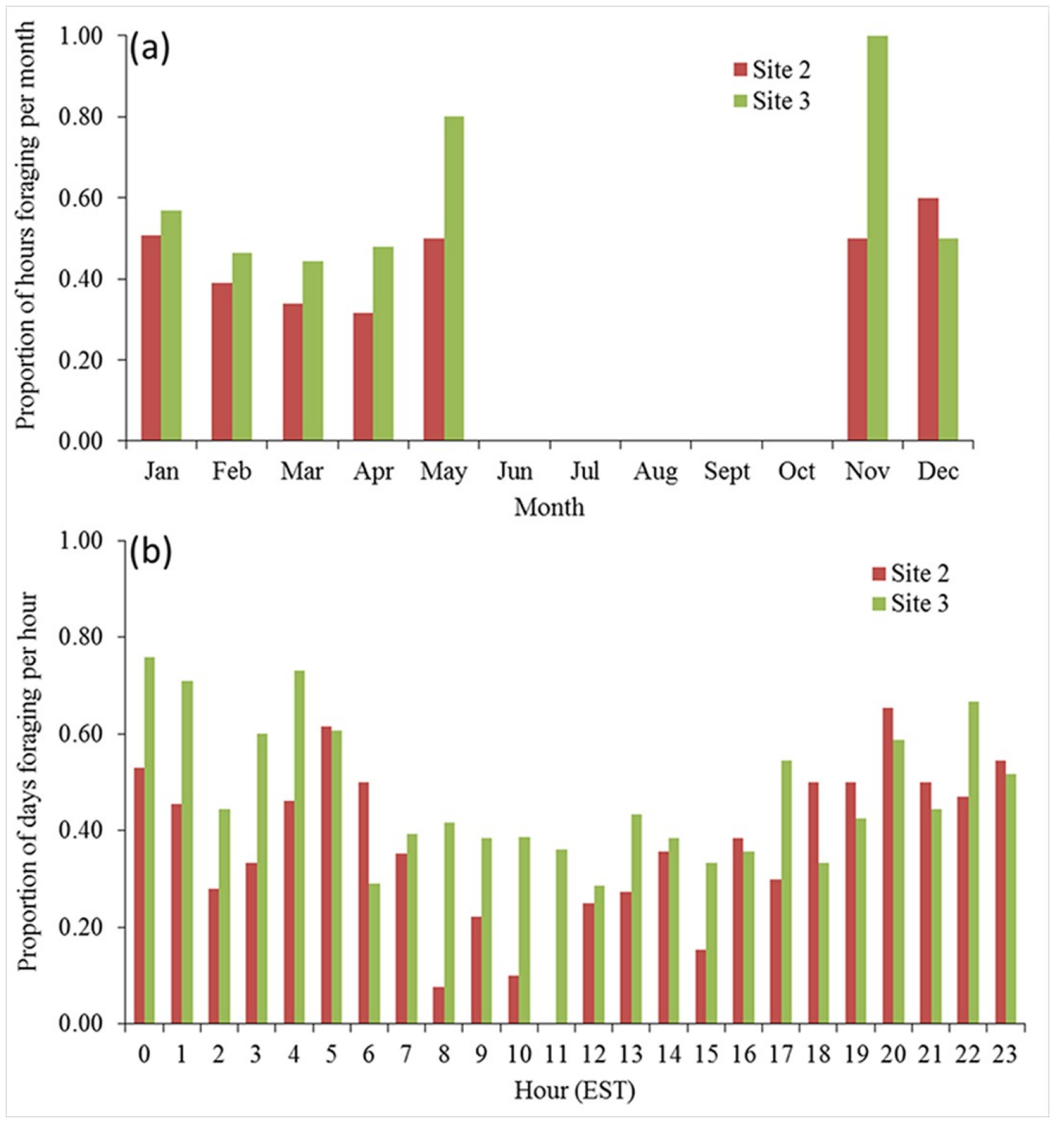
Summary of harbour porpoise foraging behaviour. The proportion of hours harbour porpoise foraging behaviour was detected in each month (a) and the proportion of days that harbour porpoise foraging was detected in each hour (b).

Although we deemed hours with at least one ICI equal to or less than 10 ms as foraging positive hours, an average of 94% of foraging hours analyzed for sites 1 to 3 contained at least 5 ICIs less than 10 ms. The occurrence of foraging at site 4 was not modeled because harbour porpoises were present for only 12 hours in total and were identified as foraging for 6 of those hours at that site in April, May and October 2015, and January 2016. In contrast, harbour porpoises foraged during 61% of the hours they were present at site 3 from November to May (Fig 5). Julian day and hour of the day were both retained in the final models for sites 1, 2, and 3 (Table 2). Both of the variables had a significant relationship with the foraging behaviour of harbour porpoises at sites 2 and 3 (Table 2). At site 2, the proportion of hours during which foraging activity occurred decreased from January (0.51) to April (0.32), before rising again in May (0.50) (Fig 5). At site 3, foraging activity decreased from January (0.57) to March (0.44), and began to increase again in April (0.48) and May (0.80) (Fig 5). The high proportion of foraging activity in November at site 3 (1.00) is based on only one hour of data when porpoises were detected. Diel patterns in foraging varied between sites (S1 Fig). A decline in foraging during daytime hours was most pronounced at site 2, where the occurrence of foraging was lowest from 08:00 to 11:00 and highest in the evening to early morning (Fig 5). All models correctly predicted the presence of foraging behaviour greater than 50% of the time, and areas under the ROC curves were all greater than 0.60.

**Table 2:** The results of the binomial generalized additive models (GAM) used to relate presence/absence of foraging to hour of the day (EST) and Julian day at sites 1, 2, and 3.

**Site 1**				
**Parametric coefficients**				
	**Estimate**	**Standard Error**	**z**	**P**
Intercept	-0.58	0.10	-5.70	<0.001
**Smooth terms**				
	**Estimated degrees of freedom**	**Reference degrees of freedom**	**Chi Square**	**P**
Hour	5.90	8	11.91	0.05
Julian Day	6.27	8	11.64	0.07
R^2^ = 0.04, deviance explained = 5.22%				
**Site 2**				
**Parametric coefficients**				
	**Estimate**	**Standard Error**	**z**	**P**
Intercept	-0.46	0.11	-4.04	<0.001
**Smooth terms**				
	**Estimated degrees of freedom**	**Reference degrees of freedom**	**Chi Square**	**P**
Hour	5.24	8	23.96	<0.001
Julian Day	2.15	8	6.27	0.03
R^2^ = 0.08, deviance explained = 7.36%				
**Site 3**				
**Parametric coefficients**				
	**Estimate**	**Standard Error**	**z**	**P**
Intercept	-0.09	0.08	-1.15	0.24
**Smooth terms**				
	**Estimated degrees of freedom**	**Reference degrees of freedom**	**Chi Square**	**P**
Hour	2.48	8	24.08	<0.001
Julian Day	3.64	8	9.59	0.02
R^2^ = 0.05, deviance explained = 4.39%				

### Association with environmental variables

**Fig 6:**
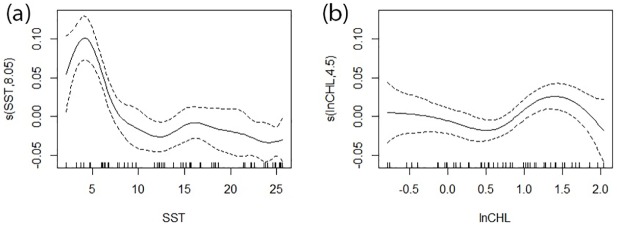
Smoothers from the generalized additive model (GAM) for site 2. The relationship between the proportion of hours per week that harbour porpoises were detected and (a) sea surface temperature (SST, °C) and (b) the natural logarithm of chlorophyll *a* concentration (mg m^-3^). The predictor is on each x-axis, the centered fitted values are on each y-axis, the dashed lines are error bands. Tick marks on the x-axes—rug plot—show the distribution of the underlying data. Similar smoother patterns occurred for sites 1 and 3.

The weekly proportion of hours harbour porpoises were present was significantly affected by SST at sites 1, 2, and 3, and by chlorophyll *a* concentration at sites 2 and 3 (Table 3). Harbour porpoises were present more often when SST was low at all three sites, with a peak in occurrence at approximately 5°C (Fig 6). At sites 2 and 3, harbour porpoises were present most often when the chlorophyll *a* concentration was approximately 4.5 to 7.4 mg m^-3^ (Fig 6). Data from site 4 were not modeled due to a high number of missing weeks and low number of detections.

**Table 3:** The results of the generalized additive models (GAM) used to relate the weekly occurrence of harbour porpoises to sea surface temperature and the natural logarithm of chlorophyll *a* concentration at sites 1, 2, and 3.

	**Site 1**			
**Parametric coefficients**				
	**Estimate**	**Standard Error**	**t**	**P**
Intercept	0.03	0.00	8.97	<0.001
	**Smooth terms**			
	**Estimated degrees of freedom**	**Reference degrees of freedom**	**F**	**P**
SST	4.76	5.82	7.83	<0.001
R^2^ = 0.36, deviance explained = 39.70%				
	**Site 2**			
**Parametric coefficients**				
	**Estimate**	**Standard Error**	**t**	**P**
Intercept	0.03	0.00	7.65	<0.001
	**Smooth terms**			
	**Estimated degrees of freedom**	**Reference degrees of freedom**	**F**	**P**
SST	8.05	8.73	10.07	<0.001
ln(Chla)	4.50	5.53	2.58	0.03
R^2^ = 0.72, deviance explained = 78.00%				
	**Site 3**			
**Parametric coefficients**				
	**Estimate**	**Standard Error**	**t**	**P**
Intercept	0.04	0.01	7.23	<0.001
	**Smooth terms**			
	**Estimated degrees of freedom**	**Reference degrees of freedom**	**F**	**P**
SST	8.19	8.80	16.42	<0.001
ln(Chla)	6.72	7.83	2.70	0.01
R^2^ = 0.77, deviance explained = 82.10%				

SST, sea surface temperature (°C); ln(Chla), natural log of chlorophyll *a* concentration.

### Comparison of acoustic data with habitat-based density predictions

**Fig 7:**
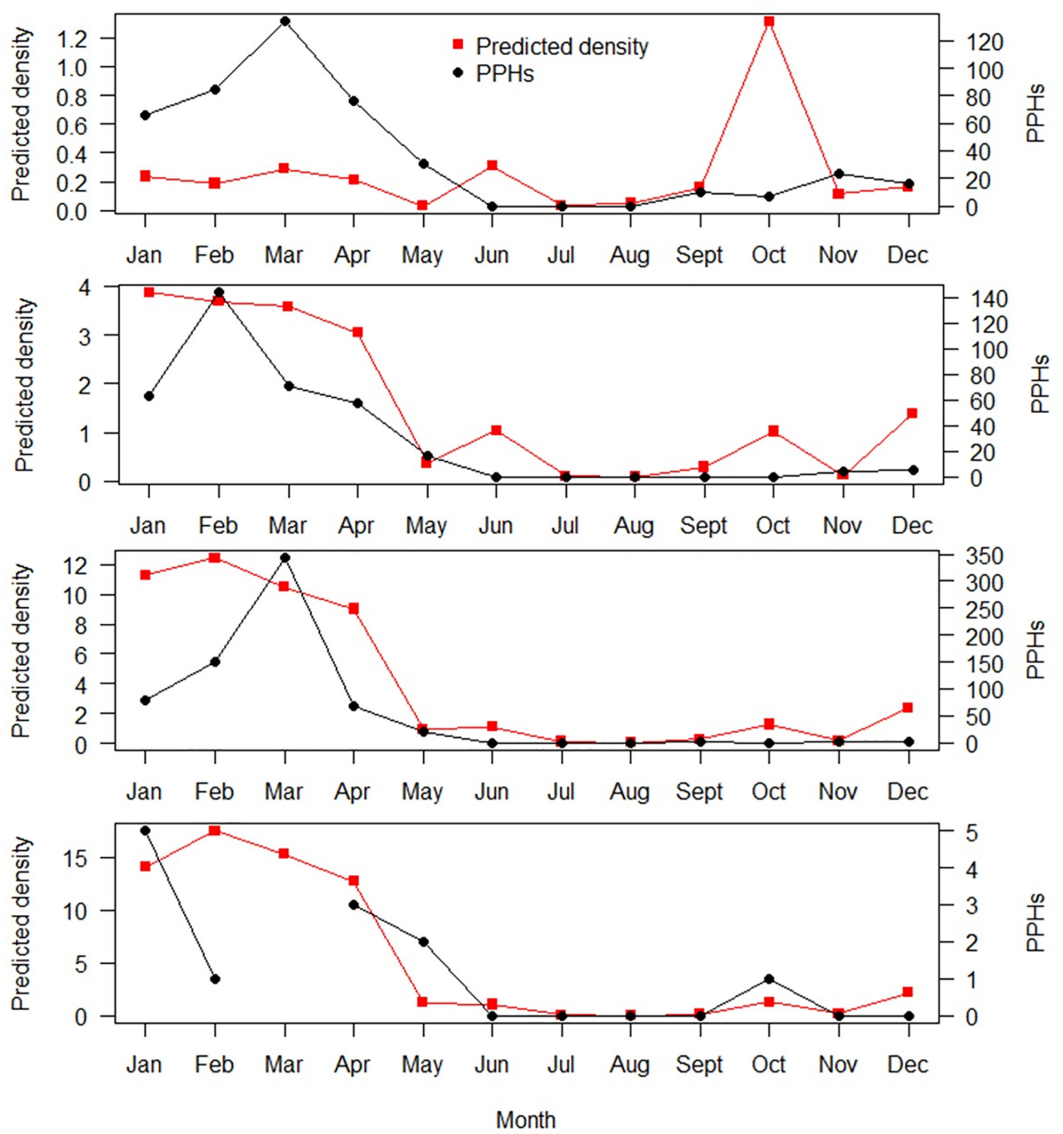
The predicted densities of harbour porpoises per month (red) and the total number of acoustically detected porpoise positive hours (PPHs) per month offshore of Maryland (black). Predictions (in individuals per 100 km^2^) are from Roberts et al.’s [25] model and acoustic data were collected from November 2014 to May 2016. There were no acoustic data for March at site 4.

Acoustic detections of harbour porpoises (median PPHs per day, total PPHs, maximum number of PPHs per day, and proportion of days per month detected) were significantly correlated with monthly habitat-based density estimates [25] at sites 2, 3, and 4 (Table 4). The median number of PPHs per day at site 4 was 0 in all months, and therefore there is no correlation value for this metric. The strongest correlations were between predicted densities and the total number of PPHs at site 2 (Fig 7), and the median number of PPHs per day at site 3 (Table 4). None of the acoustic detection metrics from site 1 were significantly correlated with the density estimates (Table 4). The highest predicted density of harbour porpoises at this site occurred in October [25], a month during which there were very few acoustic detections (Fig 7).

**Table 4:** Spearman’s rank correlation coefficients (*p*-values are in parentheses) for the median porpoise positive hours (PPHs) per day, total PPHs per month, maximum number of PPHs per day and proportion of days harbour porpoises were detected acoustically in each month compared to Roberts et al.’s [25] monthly predictions of porpoise density at each site.

Acoustic Metric	Site 1	Site 2	Site 3	Site 4
Median PPHs	0.26 (0.41)	0.59 (0.04)	0.80 (0.00)	NA
Total PPHs	0.20 (0.54)	0.80 (0.00)	0.79 (0.00)	0.73 (0.01)
Max. PPHs	0.23 (0.48)	0.78 (0.00)	0.76 (0.00)	0.74 (0.01)
Proportion	0.20 (0.52)	0.78 (0.00)	0.77 (0.00)	0.74 (0.01)

## Discussion

Harbour porpoises were regularly detected offshore of Maryland during the winter and spring, particularly from January to May. This is in contrast to low sighting rates during many boat-based and aerial surveys conducted over many years [23, 24]. Our study has shown that harbour porpoise occurrence is greatest during the winter and spring, and during hours of the day with reduced light or darkness. These are periods of time during which conditions for sighting this small species are generally poor, and visual surveys are expected to underestimate harbour porpoise occurrence.

The observed seasonal pattern in harbour porpoise occurrence is consistent with prior information on their general distribution [18–21]. Harbour porpoises move between their summer habitat in the Bay of Fundy and Gulf of Maine to as far south as North Carolina in the winter [18–21]. Harbour porpoises in this population have been found to travel a range of distances between productive habitats, where aggregations of prey may occur [68]. Our analysis of the surface chlorophyll *a* concentration suggested March to May is a period of high primary productivity offshore of Maryland, as it is during the winter-spring phytoplankton bloom[69, 70].

There was a high degree of inter-annual variation in the number of minutes per day that porpoises were detected. The maximum periods of time between the clicks of three free-ranging, tagged harbour porpoises in Danish waters were brief (1.6, 4, and 22 minutes), demonstrating that porpoises click regularly [53]. Because of this regularity in click production, patterns in the C-POD detection rates of clicks were assumed to reflect occurrence of harbour porpoises [71]. Inter-annual variability in occurrence is also reflected in the stranding record, as 22 strandings were recorded in 2005, and only two in 2011 and 2012 on the shorelines of Virginia [72]. The change in porpoise occurrence between years could be due to a number of biological and oceanographic factors affecting the environment offshore of Maryland and in more northern foraging grounds. For example, favourable conditions in more northern foraging grounds could delay porpoise movement southwards, leading to decreased or delayed occurrence offshore of Maryland. Chlorophyll *a* concentration at site 3 was greater in 2015 compared to 2016, which is likely to have led to increased prey abundance and in turn higher porpoise occurrence at this site in 2015. Further investigation into the environmental conditions in areas beyond our study area would provide insight into which factors affect broader porpoise movement up and down the coastline from year to year. Anthropogenic noise may also influence harbour porpoise occurrence and behaviour in the area, although we were unable to measure this with the C-POD.

In addition to seasonal variation in occurrence, a particularly strong diel pattern was observed at the site within the Maryland WEA (site 2), where porpoises occurred most frequently in the evening to early morning hours. This is consistent with previous studies, in which diel patterns in porpoise echolocation rates were hypothesized to be linked to prey availability [51, 55]. As visual surveys are not conducted during these hours because of reduced visibility, it is probable that porpoise occurrence at this site will be underestimated by visual surveys. It is thus recommended that future monitoring of harbour porpoise distribution in this area be conducted using passive acoustic monitoring with moored or towed hydrophones.

Foraging behaviour was analyzed using only the hours during which harbour porpoises were detected, and therefore the dataset was unevenly spaced. Temporal autocorrelation in unevenly spaced datasets cannot be correctly assessed using standard methods, and requires non-trivial estimation techniques [73, 74]. However, as foraging often occurred in non-consecutive hours and there were sometimes long gaps in foraging occurrence, we assumed that the occurrence of foraging in an hour was independent from prior and subsequent hours with foraging and did not explicitly model the autocorrelation structure. The increase in foraging activity during nighttime hours at sites 2 and 3 is consistent with patterns observed in harbour porpoise populations around the world [50, 51, 55, 75]. The diel pattern in foraging may reflect nighttime diving behaviour or prey distribution. Porpoises occurring offshore of Maryland may increase their mean dive depth during nighttime hours, as was seen in the Bay of Fundy [76], and are therefore more likely to have been detected by the bottom-moored C-POD at night. However, there was no diel pattern in dive-depth observed in Japanese waters [77]. Herring (*Clupea harengus*), one of the main prey species for harbour porpoises in the Northwestern Atlantic [78, 79], migrate vertically in the water column at night [80, 81]. This behaviour may make herring easier to prey upon at night, leading to an increase in porpoise foraging.

The deviance explained by each of the foraging models was low (<10%), and would likely increase in subsequent models with the inclusion of information on environmental conditions and the distribution and abundance of prey species. The fine-scale spatial and temporal distributions of harbour porpoise prey, such as herring, silver hake (*Merluccius bilinearis*), and pearlsides (*Maurolicus weitzmani*) [79], are not well known as their availability to trawl surveys is low [82]. Even if trawls effectively captured forage fish, the surveys cover large areas and data are often aggregated on a seasonal scale. Sediment type has been used in harbour porpoise habitat association models as a proxy for sandeels (also known as sand lance, *Ammodytes* species) [35], a key prey species for harbour porpoises in European waters [83], which prefer fine and coarse sand sediments. Sand lance also occur in the mid-Atlantic Bight [84], and the dominant sediment type in our study area is sand. Fine-scale data on prey abundance, for example using sonar-imaging technology [85], is another way to improve our understanding of factors driving porpoise foraging behaviour.

As in previous studies (e.g. [65]), we used environmental variables as proxies for prey abundance because fine-scale data on prey were not available. Chlorophyll *a* concentration, SST and fraction of the moon illuminated were readily available data sets. Despite being a significant factor influencing the echolocation of some dolphin species [66], lunar illumination did not significantly affect harbour porpoise echolocation offshore of Maryland. SST significantly affected harbour porpoise occurrence at all three sites. This result is consistent with Roberts et al.’s [25] model, which predicted greater harbour porpoise presence at lower SSTs. Harbour porpoises were expected to be present at colder temperatures given their seasonal distribution pattern. The peak in harbour porpoise detection rate at 5°C at all sites may also relate to the presence of herring, as catches were greatest in waters of 7–8°C in winter and 5°C in spring [86]. Summertime (June to October) concentrations of chlorophyll *a* in the mid-Atlantic Bight are typically below 1 mg m^-3^ [69], compared to values exceeding 3 mg m^-3^ in coastal areas during the winter-spring bloom, which begins as early as January and continues until March or April [69, 70]. It is during this winter-spring bloom that porpoise presence peaked at sites 2 and 3, at chlorophyll *a* concentrations of 4.5 to 7.4 mg m^-3^. These values are particularly high, even for this productive period in the mid-Atlantic coastal waters. Peaks in porpoise occurrence at higher chlorophyll *a* concentrations may be linked to prey, as areas of higher primary productivity are likely to have greater numbers of forage fish [64]. Roberts et al.’s [25] final models of summer and winter harbour porpoise density also retained productivity parameters, which had positive effects on porpoise density. The models at sites 2 and 3 relating our acoustic detections to environmental variables explained a high percentage of the deviance in weekly porpoise occurrence (78.0 and 82.1% respectively), indicating SST and chlorophyll *a* concentration are appropriate indicators for porpoise occurrence offshore of Maryland. The inclusion of tidal parameters may help to improve model fit for site 1 occurrence, where the deviance explained was low [32, 87].

All of the acoustic metrics for sites 2–4 were significantly correlated with monthly habitat-based predictions of harbour porpoises from sightings data recorded during aerial and boat-based surveys [25]. However, the monthly density predictions for site 1 did not correlate well with the acoustic data. Roberts et al. [25] fit two separate models, one for the winter (November to May) and another for the summer (June to October) data, as it was assumed porpoises switch environmental preferences during different phases of their annual migratory cycle. Although this strategy worked well when modelling baleen whale occurrence, it resulted in a rise in porpoise density at the May to June transition and discontinuity at the October to November transition, which was most evident at site 1. The results from this study can be used to inform how to refine and improve the density models. Although it is difficult to determine absolute densities of cetacean species using passive acoustic data [43, 88], this type of data can be a useful, independent data source to validate relative patterns and improve habitat-based models.

This study provides insight into the previously poorly understood occurrence of harbour porpoises offshore of Maryland and indicates that it is underestimated when using boat-based and aerial survey methods. The diel pattern in detections can be used to improve estimates of the detection probability for harbour porpoises during line transect surveys. Harbour porpoises occurred frequently offshore of Maryland from January to May. Consistent with our findings on their seasonal occurrence in the southern part of their range, strandings of porpoises after entanglement in fishing nets occurred primarily from January to May along the shores of Maryland, Virginia, and North Carolina [72, 89]. Scheduling wind farm construction activities in the Maryland WEA to take place during the summer months (June to September) would reduce the likelihood of disturbance to harbour porpoises. However, there are many other protected species that occur in the area, including the endangered North Atlantic right whale (*Eubalaena glacialis*) and endangered Atlantic sturgeon (*Acipenser oxyrinchus*), which should also be considered.

## Supporting information

S1 Fig The estimated relationships between the presence/absence of foraging and hour of the day (Eastern standard time, EST) at sites 1, 2, and 3. (TIF)

S1 TableEstimated parameters (standard errors in parentheses) from the generalized auto-regressive moving average (GARMA) models used to relate the number of minutes harbour porpoises were present in an hour to the hour (EST) and Julian day.Explanatory variables were sine and cosine transformed to capture the daily and seasonal cycles. PIG = Poisson inverse-Gaussian, ZIP = Zero-inflated Poisson. Asterisks represent significance level: 0 ‘***’ 0.001 ‘**’ 0.01 ‘*’ 0.05 ‘.’ 0.1 ‘ ‘ 1. The general formula for a GARMA model is:
$$g\left(\mu_{t}\right)=X_{t}^{\text{'}}\beta +\Sigma_{j=1}^{p}\varphi_{j}\left\{\text{g}\left(Y_{t-j}\right)-X_{t-j}^{\text{'}}\beta \right\}+\Sigma_{j=1}^{q}\theta_{j}\left\{\text{g}\left(Y_{t-j}\right)–\text{ g}\left(\mu_{t-j}\right)\right\},$$(1)
where *g*(·) is the link function, *μ*_t_ is a conditional mean of the dependent variable, *β* is the regression coefficients, *φ*_j_ and *θ*_j_ are the autoregressive and moving average parameters, and *p* and *q* are the orders, respectively [1, 2].(DOCX)
