# Supplementary figures and images for: Year-round spatiotemporal distribution of harbour porpoises within and around the Maryland wind energy area
Source: PLoS One. 2017 May 3;12(5):e0176653. doi: 10.1371/journal.pone.0176653 (PMC5415022; doi:10.1371/journal.pone.0176653)

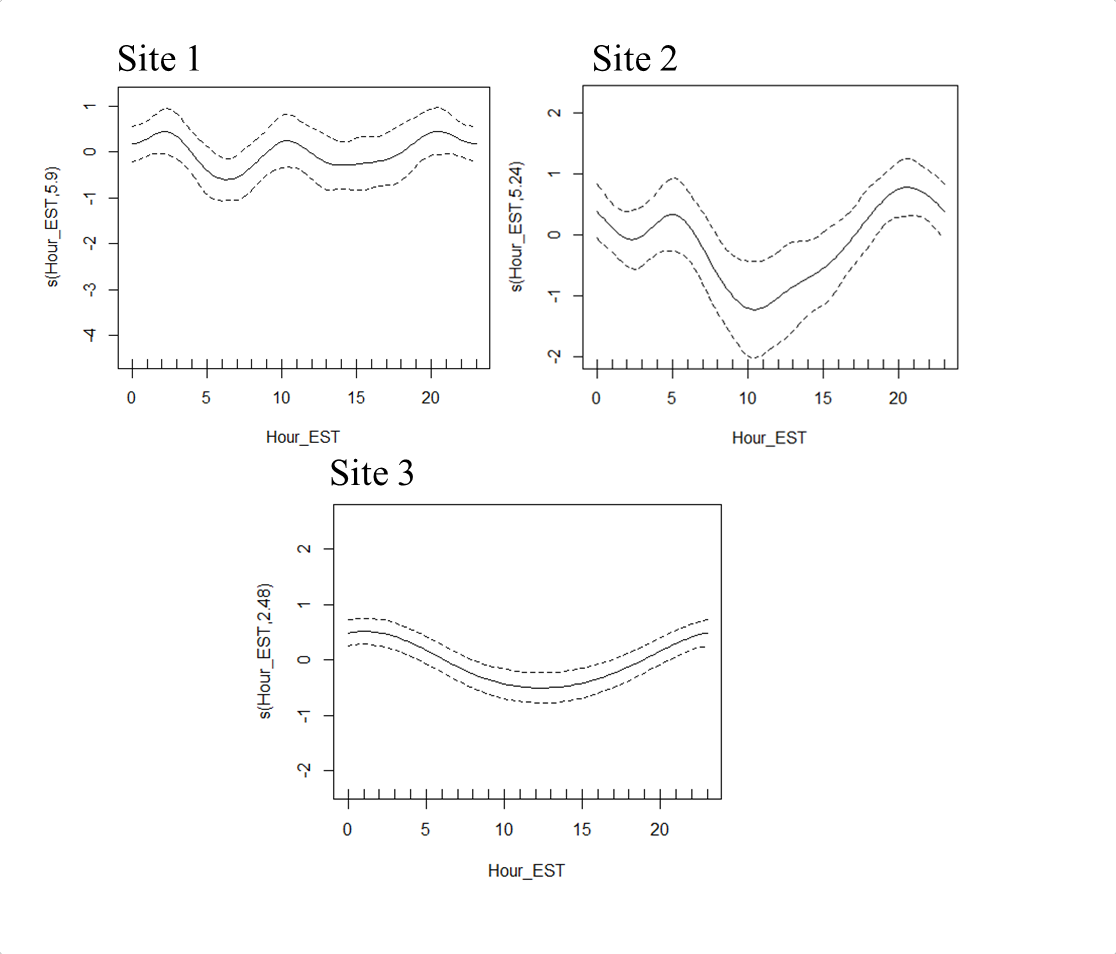

Supplement: S1 Fig — (TIF) [file pone.0176653.s001.tif]
