# Supplementary material for: Year-round spatiotemporal distribution of harbour porpoises within and around the Maryland wind energy area
Source: PLoS One. 2017 May 3;12(5):e0176653. doi: 10.1371/journal.pone.0176653 (PMC5415022; doi:10.1371/journal.pone.0176653)
Supplement: S1 Table — Explanatory variables were sine and cosine transformed to capture the daily and seasonal cycles. PIG = Poisson inverse-Gaussian, ZIP = Zero-inflated Poisson. Asterisks represent significance level: 0 ‘***’ 0.001 ‘**’ 0.01 ‘*’ 0.05 ‘.’ 0.1 ‘ ‘ 1. The general formula for a GARMA model is: g(μt)=Xt'β+Σj=1pφj{g(Yt−j)−Xt−j'β}+Σj=1qθj{g(Yt−j)– g(μt−j)},(1) where g(·) is the link function, μt is a conditional mean of the dependent variable, β is the regression coefficients, φj and θj are the autoregressive and moving average parameters, and p and q are the orders, respectively [1, 2]. (DOCX) [file pone.0176653.s002.docx]

| Site | Distribution | β_Intercept_ | β_sinhour_ | β_sinday_ | β_cosday_ | β_coshour_ | θ_1_ | θ_2_ | θ_3_ | θ_4_ | φ_1_ |
| --- | --- | --- | --- | --- | --- | --- | --- | --- | --- | --- | --- |
| 1 | PIG | -6.250*** (0.461) | 0.698* (0.302) | 5.280*** (0.594) | 3.175*** (0.471) | - | 0.841*** (0.073) | 0.591*** (0.097) | 0.361*** (0.091) | 0.233** (0.081) | - |
| 2 | PIG | -7.523*** (0.517) | - | 7.414*** (0.704) | 3.028*** (0.407) | 1.030*** (0.218) | 0.506*** (0.068) | 0.297*** (0.062) | - | - | - |
| 3 | PIG | -83.119*** (14.327) | - | 107.413*** (19.043) | 41.216*** (7.084) | - | -0.374*** (0.048) | -0.176*** (0.049) | - | - | 0.976*** (0.005) |
| 4 | ZIP | Intercept | Sinday |  |  |  |  |  |  |  |  |
|  |  | -1.448** (0.448) | 2.728*** (0.565) |  |  |  |  |  |  |  |  |

**References**

1. Benjamin MA, Rigby RA, Stasinopoulos DM. Generalized autoregressive moving average

models. J Am Stat Assoc. 2003;98(461):214-23. doi: 10.1198/016214503388619238.

2. Soliman M, Lyubchich V, Gel YR, Naser D, Esterby S. Evaluating the impact of climate

change on dynamics of house insurance claims. In: Lakshmanan V, Gilleland E, McGovern A, Tingley M, editors. Machine Learning and Data Mining Approaches to Climate Science. Switzerland: Springer International Publishing; 2015. p. 175-83.
